# Lipid-Based Nanosystems as a Tool to Overcome Skin Barrier

**DOI:** 10.3390/ijms22158319

**Published:** 2021-08-02

**Authors:** Maddalena Sguizzato, Elisabetta Esposito, Rita Cortesi

**Affiliations:** 1 Department of Chemical, Pharmaceutical and Agricultural Sciences (DoCPAS), University of Ferrara, I-44121 Ferrara, Italy; sgzmdl@unife.it (M.S.); ese@unife.it (E.E.); 2 Biotechnology Interuniversity Consortium (C.I.B.), Ferrara Section, University of Ferrara, I-44121 Ferrara, Italy

**Keywords:** liposomes, cubosomes, monoolein, transferosomes, ethosomes, SLN, NLC, skin

## Abstract

Skin may be affected by many disorders that can be treated by topical applications of drugs on the action site. With the advent of nanotechnologies, new efficient delivery systems have been developed. Particularly, lipid-based nanosystems such as liposomes, ethosomes, transferosomes, solid lipid nanoparticles, nanostructured lipid carriers, cubosomes, and monoolein aqueous dispersions have been proposed for cutaneous application, reaching in some cases the market or clinical trials. This review aims to provide an overview of the different lipid-based nanosystems, focusing on their use for topical application. Particularly, biocompatible nanosystems able to dissolve lipophilic compounds and to control the release of carried drug, possibly reducing side effects, are described. Notably, the rationale to topically administer antioxidant molecules by lipid nanocarriers is described. Indeed, the structural similarity between the nanosystem lipid matrix and the skin lipids allows the achievement of a transdermal effect. Surely, more research is required to better understand the mechanism of interaction between lipid-based nanosystems and skin. However, this attempt to summarize and highlight the possibilities offered by lipid-based nanosystems could help the scientific community to take advantage of the benefits derived from this kind of nanosystem.

## 1. Introduction

It is well known that skin is an important body barrier towards external chemical, mechanical, physical, and microbial stresses, resulting in a protection against pathogens and water loss [1,2,3]. Notwithstanding these important functions, skin may be affected by many problematic disorders (e.g., rashes), infections of different nature (i.e., viral, bacterial, fungal, and parasitic), injuries caused by cuts or burns, and tumors [4,5,6,7]. The treatment of skin disorders can be done with topical applications of drugs on the action site. In this way, the drug is less involved in causing systemic side effects than with systemic application, since the drug barely reaches the systemic circulation. When the affected area requires a high concentration of a drug, intradermal injection may be considered, but the use of more appropriate administration strategies to treat systemic diseases may be more beneficial and effective [8,9]. The success of topical treatments may depend on the type of vehicle used to deliver the drug to the skin, and also on the method of administration.

Indeed, the vehicle is important to give texture to the formulation and also to drive drug penetration through the skin. It is known that the drug can be included in different types of semi-solid formulation, such as pastes, creams, gels, oils, and ointments; however, in clinical practice, these formulations can cause problems of poor skin penetration and localized side effects [10,11]. Therefore, in recent years, with the advent of nanotechnologies, it has been possible to respond to the necessity of developing more efficient drug delivery systems by trying to overcome the described drawbacks and to implement skin release [12,13,14]. With this in mind, the design and production of nanoscale material for technological applications, such as lipid-based nanosystems, can lead to benefits in the treatment of skin diseases [15,16].

Particularly, lipid-based nanosystems such as liposomes [17,18], ethosomes [19,20], transferosomes, solid lipid nanoparticles (SLNs) [21,22], nanostructured lipid carriers (NLCs), cubosomes, and monoolein aqueous dispersions [23,24] have been proposed for cutaneous application, reaching in some cases the market or clinical trials. Indeed, these biocompatible lipid-based nanosystems allow the dissolution of lipophilic compounds, therefore increasing their bioavailability, and ability to control the release of carried drug, possibly reducing side effects. In addition, the application onto the skin of food-grade lipids creates an emollient effect with low onset of irritation or allergic events [25,26].

Furthermore, the structural similarity between the lipids composing the nanosystems and those of the skin represents one of the main advantages of lipid-based nanosystems, allowing the interaction between the nanosystem matrix and the *stratum corneum* [27,28], therefore increasing skin hydration and, possibly, also the penetration of the carried active molecule [29,30,31].

This review provides an outline of the different lipid-based nanosystems and focuses on their use for topical application of drugs. In particular, the application of active molecules with antioxidant activity is considered.

## 2. Lipid-Based Nanosystems

Natural lipids are widespread molecules that are usually highly biodegradable, low-toxic, and easily handled during lipid-based nanosystems (LBNs) production procedures that play an important role for living cell processes [21,25].

**Figure 1:**
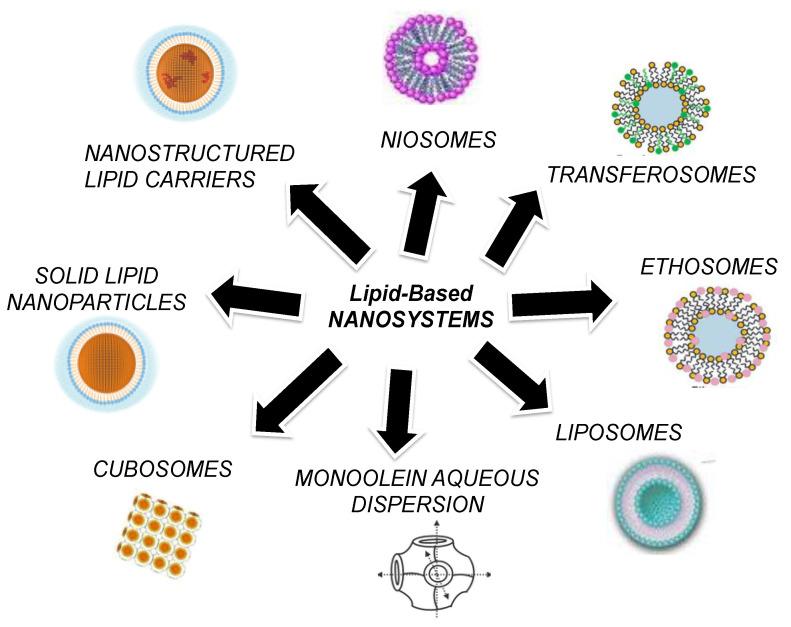
Schematic representation of LBNs considered in this review.

Among the LBNs, it is possible to find many kinds and categories. Indeed, the architecture of the nanosystem can be quite different depending of the type of constituent or the production method chosen. Particularly, nanoemulsions, lipid-core micelles, vesicles such as liposomes, ethosomes, transethosomes, or niosomes, and also nanoparticles, including SLNs, NLCs, cubosomes, and monoolein-based systems, can be listed as LBNs. Figure 1 schematically summarizes the main LBNs considered in this review.

### 2.1. Liposomes

**Figure 2:**
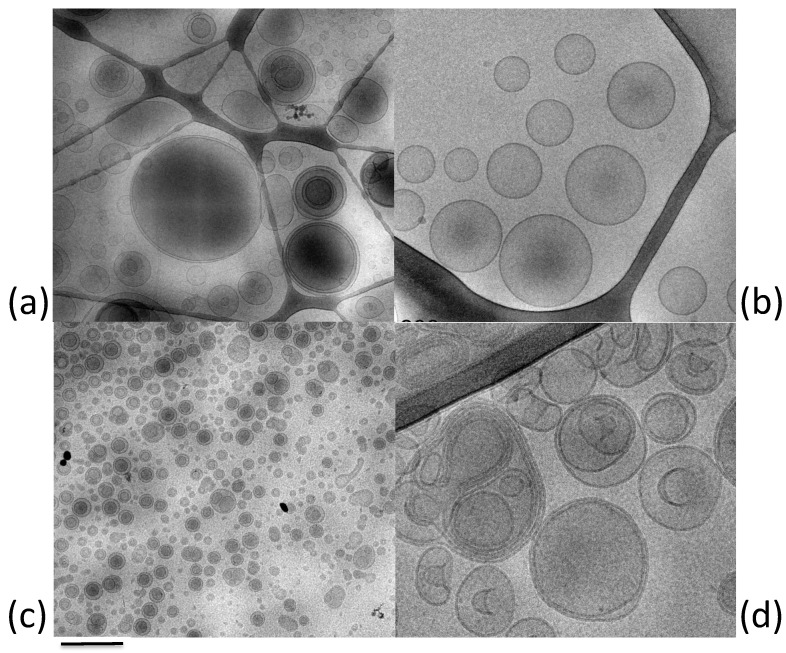
Cryo-TEM images of exemplary LBNs cited in this review: (**a**) liposomes; (**b**) transferosomes; (**c**) niosomes; (**d**) ethosomes. Bar corresponds to 500 nm (**a**,**c**) and 200 nm (**c**,**d**).

Liposomes are vesicular delivery systems that were first described by Bangham [32,33]. They are composed of amphiphilic molecules arranged spontaneously in a spherical, closed bilayer structure when in aqueous solution (Figure 2a). Hence, this organization promotes the delivery of both hydrophilic and lipophilic molecules considering the presence of compartments with different hydrophobic grades [34]. They can be classified as unilamellar or multilamellar according to the formulation method. The preparation process influences the size and the lamellarity, resulting in unilamellar liposomes of between 30 and 500 nm and multilamellar liposomes of >500 nm [35].

Thanks to the nature of the major components used in the formulation, such as phospholipids (phosphatidyl choline or lecithin, phosphatidyl ethanolamine or cephalin, phosphatidyl serine, phosphatidyl inositol, and phosphatidyl glycerol) and cholesterol [36], liposomes can be considered biocompatible, biodegradable, and nontoxic delivery systems. Moreover, the similarity of phospholipids to biological membranes and the stabilizing effect of cholesterol ensure their excellent interaction with the molecular target [37].

Liposomes have been widely explored, and a great number of formulations are now in clinical use in different application fields, as reported by several authors [33,34,38]. Nevertheless, their therapeutic potential is limited by some drawbacks related to the tendency of phospholipids oxidation, the presence of organic solvents in the formulation stage, the poor storage stability, the drug leakage, the rapid systemic clearance, and the limited malleability [33,39,40].

For these reasons, liposomes have been innovated, and several modifications in their composition resulted in new generations of vesicular systems.

### 2.2. Transfersomes

Transfersomes were introduced in 1992 [41,42] with the aim of developing liposomes with high deformability, elasticity, and ultra-flexibility. The typical characteristic of these vesicles is determined by the addition to phospholipids of a single-chain surfactant as an edge-activator [35,43] that is able to reduce the interfacial tension, leading to a destabilization in the lipid bilayers [43,44]. Therefore, the insertion of edge-activator between the phospholipids generates discontinuities in the structure, resulting in ultra-deformable vesicles (Figure 2b) [45]. The major edge-activators used to produce transfersomes are sodium cholate, sodium deoxycholate, Span (namely, 60, 65, and 80), Tween (namely, 20, 60, and 80), dipotassium glycyrrhizinate, and polysorbic acid [46,47].

**Figure 3:**
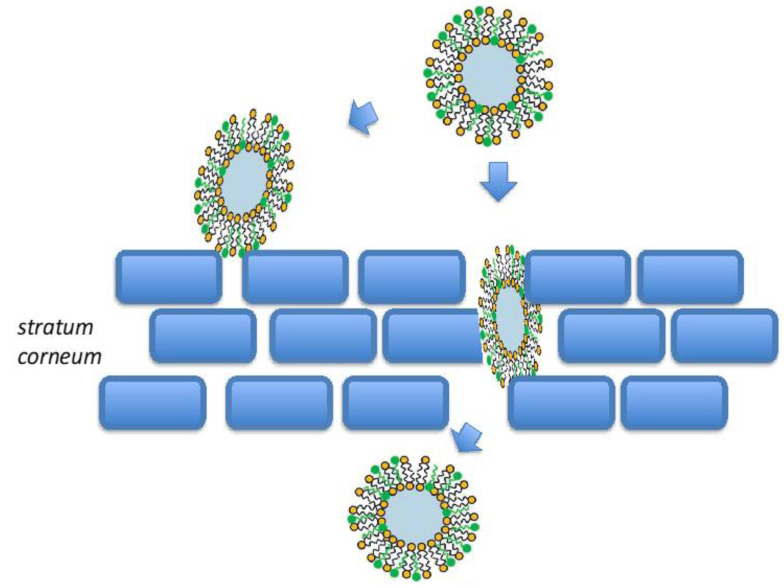
Schematic representation of transfersome penetration through the epidermis.

Transfersomes have the same advantages of liposomes, thanks to the inner aqueous compartment surrounded by lipid bilayers and the biocompatibility of the components. Moreover, their development improved the efficiency of vesicular systems in topical application [48]. The elastic behavior of transfersomes given by the presence of ellagic acid improves their penetration through the skin, allowing the transdermal delivery of encapsulated drugs, when in non-occlusive conditions [38,49]. The main mechanism related to the skin penetration of transfersomes is the theory of osmotic force [50]. There are three major pathways for molecules to pass the *stratum corneum,* namely, intercellular, intracellular, and follicular. The first one is via a tortuous path through the lipids surrounding the corneocytes, while the second one is more restrictive, as molecules must pass through both corneocytes and the intercellular lipids. Finally, the third path (follicular route) by using hair shafts and sebaceous glands allows the penetration of molecules through hair follicles openings [51,52]. However, the passage across the skin of these highly deformable (ultra-flexible) transfersomes is mainly attributable to the membrane flexibility, hydrophilicity, and the ability to maintain the vesicle’s integrity (as schematized in Figure 3) [50,53,54]. Particularly, the different layers of the skin possess different water content, generating a hydrostatic gradient in the skin [35,55]. The upper layers are less hydrated and practically dry compared to the lower epidermal layers, thus, after topical administration, transfersomes are driven by the water gradient in the deeper skin layers [45,56].

Furthermore, thanks to their own deformable nature, their possible effectiveness as transdermal delivery systems is not only related to the passage through the skin layers even up to the dermis, but also to the achievement of systemic circulation [34,57].

In addition to the research on skin delivery, transfersomes have been investigated also for the encapsulation of different therapeutics, such as antimicrobial, anticancer, antioxidant, and anti-inflammatory molecules, proteins, vaccines, and anesthetics, by exploiting different administration routes [33,43,45]. Nowadays, two transfersomes-based products are commercialized [58], but challenges are still focused on the high cost and scalability processes, phospholipids degradation, limited occlusive administration, and hydrophobic encapsulation that may affect the vesicles elasticity [44,56].

### 2.3. Niosomes

Niosomes have been firstly developed for cosmetic use, then these vesicles have been explored in pharmaceutical field thanks to their promising potential as drug delivery systems [59,60]. Niosomes are structurally similar to liposomes, being bilayered unilamellar or multilamellar vesicles composed of self-assembled non-ionic surfactants in an aqueous medium (Figure 2c) [61]. Though mainly formed by non-ionic surfactants, niosomes sometimes include cholesterol as a stabilizer. The employment of non-ionic surfactants mainly benefits both topical and transdermal delivery since they are considered nontoxic and non-irritant as compared to both cationic and anionic surfactants [62,63]. The amphiphilic nature of the system ensures the main advantages reported for liposomes, such as the encapsulation of both hydrophilic and hydrophobic drugs as well as their biodegradability [33,48,59]. However, the presence of non-ionic surfactants instead of lipids guarantees the physical and chemical stability of the system and easy formulation with low-cost and large-scale production [59,64]. In fact, a serious inconvenience of liposomes is typified by the hydrolysis, oxidation, and rancidity of phospholipids used, which affect the storage and the bioavailability of drug. The use of appropriate surfactants, selected on the basis of the hydrophilic–lipophilic balance (HLB) and critical packing parameter (CPP) values, leads to successful niosomes formulation [61]. In particular, HLB facilitates the selection of oil-soluble or water-soluble surfactant, when its value is less than 9 or higher than 11, respectively. Additionally, CPP predicts the type of niosomes, namely, spherical, nonspherical, bilayer, or inverted vesicles [61]. The major non-ionic surfactants used for niosomes formulation are Span, Tween, and Brij [57,61], in addition to ester-linked surfactants, glucosyldialkyl ethers, polyglycerol alkyl ethers, crown ethers, and polyoxyethylene alkyl ethers [37].

A comparison study between liposomes and niosomes as carriers to improve the transdermal bioavailability of *Annona squamosal* extract performed by Mohamad and Fahmy [65] demonstrated the potential of both carriers to penetrate the outer layer of the skin and that niosomes and liposomes lasted for a long time in the skin but niosomes demonstrated a longer time of drug release through the skin [65]. These results confirmed a behavior obtained previously by Fang and colleagues [66] demonstrating for niosomes a higher stability after 48 h incubation compared with liposomes [66]. In addition, a study conducted by the group of Karami to deliver superoxide dismutase to hair follicles for treating alopecia areata showed that niosomes preserved enzyme activity, of which 90% was encapsulated into labrafil niosomes. Moreover, niosomes increased the affinity of superoxide dismutase permeated through the skin mainly by a follicular pathway, representing the main site of action in alopecia areata [67].

Apart from cosmetic application, niosomes have been studied for the encapsulation of numerous drugs, such as anticancer, antioxidant, diabetic therapy, anti-inflammatory, antimicrobial, anti-Alzheimer, and vaccines [48,59,61], thanks to the wide range of administration routes exploited by these types of vesicles. In fact, the versatile behavior of niosomes ensures a great pharmaceutical potential of the oral, transdermal, ocular, intravenous, vaginal, and pulmonary delivery routes [61]. For instance, in a study conducted by Lu and colleagues [68], it was demonstrated that niosomes improved the penetration of quercetin into the epidermis as compared to the corresponding solution of the free drug. Nevertheless, some physical instability phenomena described for liposomes have also been found in niosomes; therefore, limited niosomal formulations have been extended to the market [33].

### 2.4. Ethosomes

A novel type of elastic vesicular system is represented by ethosomes developed and described by Touitou [69] (Figure 2d). The peculiar characteristic of ethosomes is the presence of ethanol in the composition. Particularly, an ethanol solution of phosphatidylcholine is added to an aqueous solution, generating multilamellar vesicles [40]. Ethanol, reaching high concentrations of up to 45%, provides a soft and malleable structure to vesicles, decreases their size, and increases their stability over time and the entrapment efficiency of either hydrophilic and lipophilic drugs [35,70]. Moreover, it donates a negative surface charge to the system, preventing the vesicles aggregation and drug leakage [56].

The flexible behavior of vesicles is due to the interaction between ethanol and phosphatidylcholine. Actually, the solvent decreases the phase transition temperature of phosphatidylcholine by replacing the hydrophilic head group and consequently, it promotes the passage from gel state to high elastic liquid crystalline phase [44,56].

As transfersomes, ethosomes have been largely investigated in transdermal delivery. Indeed, the most important feature of ethanol in the formulation is the ability to act as penetration enhancer, promoting the skin permeation in depth or directly into systemic circulation. Firstly, ethanol increases the lipid membrane permeability by disturbing the organization of the *stratum corneum*. Then, the elastic nature of vesicles enables their passage through the disturbed *stratum corneum,* creating their own pathways [70]. Finally, the fusion of ethosomes in deeper skin layers generates the release of drug with transdermal absorption [71]. For instance, Sguizzato et al. [72] demonstrated the suitability of ethosomes as a transcutaneous delivery system for coenzyme Q10, a lipophilic endogenous antioxidant involved in the production of cellular energy. Particularly, the study confirmed the potential of these LBNs in solubilizing and delivering the lipophilic drug, acting as transdermal delivery system.

It should be noticed that the presence of ethanol seems to have a significant role on the permeation flux; thus, the larger the ethanol content, the greater the permeation flux [73]. Notwithstanding, the follicular route of skin penetration has been proposed as a possible mechanism of ethosomes delivery; therefore, the exact process of transdermal delivery is still under investigation [52].

Compared to the other vesicular systems, ethosomes possess multiple advantages, such as effectiveness in occlusive and non-occlusive conditions, ultra-flexible structure, encapsulation of water soluble and nonsoluble drugs, enhanced dermal, transdermal, intracellular delivery, higher stability, smaller vesicle size, good compliance, and multidisciplinary application [35,44,70].

**Figure 4:**
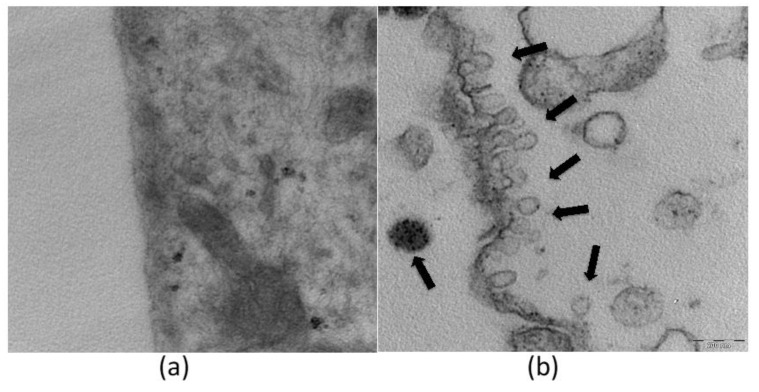
TEM images of fibroblasts untreated (**a**) and treated with ethosomes (**b**). Ethosomes (arrows) are clearly recognizable both outside and inside the cytoplasm. Bar corresponds to 200 nm.

As shown in Figure 4, TEM analysis confirmed the uptake of ethosomes within fibroblasts [74]. The morphology of the vesicles is comparable to that obtained by cryo-TEM visualization reported in Figure 2, where an external phosphatidylcholine bilayer and an inner core are evident. It should be underlined that ethosomes maintain their own structure, and that cell organelles were not altered after the entrance of ethosomes into the cytoplasm. These findings are consistent with previous studies showing that ethosomes are able to enter through the cellular membrane and release the carried molecule within the cell [71,75,76,77,78].

Some ethosomal formulations are currently marketed in the cosmetic field [33,56], and recent patents have been reported on ethosome delivery systems [56]. Even though some authors have reported that the residual content of ethanol in the formulation could induce possible skin irritation [56], others have demonstrated their safeness by in vivo irritation study [79]. In particular, the possible irritant reactions induced by cutaneous application of nanoparticulate systems were evaluated by a patch test performed on 20 health volunteers, demonstrating that ethosomes can be classified as not irritating if applied to human skin.

The development of elastic vesicles enhanced the transdermal delivery efficiency, maintaining the peculiar “green” properties of vesicular systems. Taking together, the highlighted advantages of transfersomes and ethosomes led to transethosomes being developed by Song and colleagues [80] to increase the permeability in a single formulation [37,38,81]. They are composed of phospholipids, water, ethanol, and edge activator, giving rise to more stable systems, capable to encapsulate larger molecules and permeate easily through the skin [81]. The improved composition of ethosomes offers significant advantages in the delivery of therapeutic agents, particularly over the conventional liposomes, regarding different pathologies, including acne, psoriasis, alopecia, skin infections, and hormonal deficiencies, among others [82].

Recently, the group of Albash proposed transethosomes for the transdermal delivery of the antihypertensive drug olmesartan medoxomil. Particularly, among fifteen compositions prepared by thin-film hydration technique on the basis of a full factorial design, an optimal formula was selected with good results in terms of morphology, drug encapsulation, and high drug release. Moreover, the elasticity measurement, the evaluation of ex vivo skin permeation performed either on rat or on snake skins, and the in vivo histopathological study confirmed the good performance of transethosomes as compared to other formulations, although further studies are needed to establish the therapeutic activity of this formulation in humans [83]. Moolakkadath and colleagues demonstrated that fisetin-loaded transethosomes formulations could be optimized using Box–Behnken design [84]. In particular, the optimized formulation was nanosized, characterized by good entrapment efficiencies, and showed better penetration across rat’s skin as compared to the control solution, indicating a good potential of this LBN as drug carrier for dermal delivery of fisetin [84]. On the other hand, Sguizzato et al. demonstrated that transethosomes and ethosomes are able to deliver the natural antioxidant mangiferin to the target cell, enhancing the keratinocyte antioxidant defense status, while protecting from the cutaneous ox-inflammatory damage induced by cigarette smoke ameliorating the drug solubility and delivery [74]. Moreover, the study showed the presence of intact vesicles of both ethosomes and transethosomes within keratinocytes, despite the need for in vivo experiments to clarify the mechanism of vesicle disaggregation within the cells and how these LBNs interact with the skin. Lastly Ascenso and colleagues [85] compared the behavior of the ultra-deformable vesicles transfersomes, ethosomes, and transethosomes for the incorporation of actives of distinct polarities (i.e., vitamin E and caffeine) and for the effect on skin permeation and penetration. They found that the release profile depends on the chemical properties of the incorporated molecule and on their incorporation efficiency within the different formulations. The flux and release profile followed the order transethosomes > ethosomes > transfersomes for both vitamin E and caffeine formulations. The obtained difference between transethosomes and the others ultra-deformable vesicles confirmed their best suitability for deeper skin penetration [85].

### 2.5. Cubosomes and Monoolein Aqueous Dispersions (MADs)

**Figure 5:**
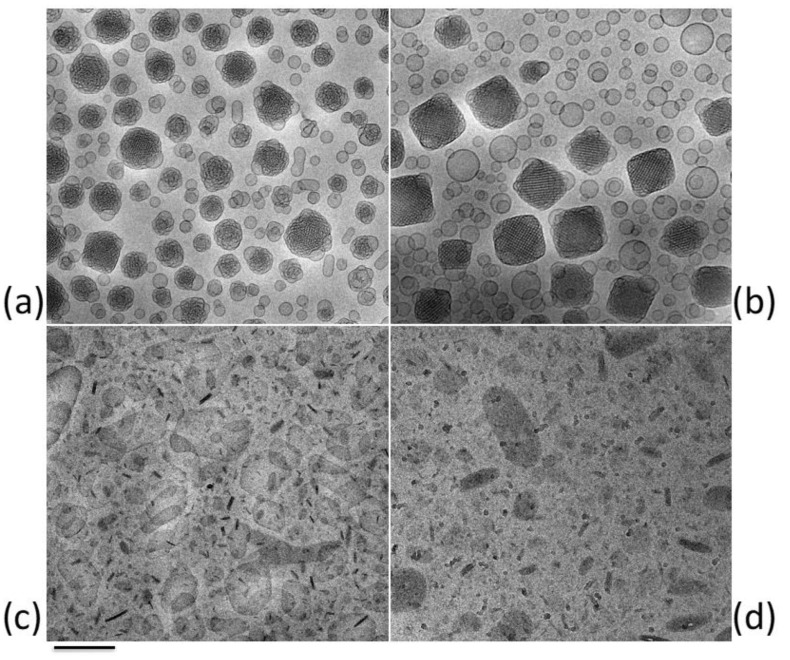
Cryo-TEM images of exemplary LBNs cited in this review: (**a**) MADs; (**b**) cubosomes; (**c**) SLNs; (**d**) NLCs. Bar corresponds to 200 nm (**a**–**c**) or 100 nm (**d**).

Cubosomes and monoolein aqueous dispersions (MADs) (Figure 5) are lipid nanosystems with optimal characteristics for delivering drugs to skin and mucosae. For instance, MADs, obtained by dispersing the unsaturated long-chain monoglycerides monoolein in water, are a mixture of heterogeneous nanosystems such as micelles and lamellar, hexagonal or cubic phases, conventionally called cubosomes [86,87], becoming an interesting system to empower solubility and control drug delivery [88]. Moreover, differently from viscous cubic phases, cubosomes and MADs being dispersed in a surfactant water solution are quite easy to handle and to administer by various routes. Indeed, these LBNs possess interesting features, such as a cubic ordered organization, biocompatibility, and bioadhesion [24]. In addition, few studies suggested a similarity between the typical ordered structure of cubosomes and that of the *stratum corneum* and highlighted the suitability of these nanosystems to promote absorption through the skin. However, despite their cubic ordered organization, these LBNs must be thickened by mean of hydrogel dilution or a colloidal polymer must be added within the dispersion in order to allow a cutaneous application [89,90,91].

Phytantriol- and glycerol monooleate-based cubosomes were produced and characterized as a targeted and sustained transdermal delivery system for capsaicin [92]. It was found that cubosomes provided sustained release of capsaicin under diffusion control. Although the percutaneous absorption of capsaicin from cubosomes was lower than that from the conventional cream, these LBNs yielded higher and sustained skin retention of the drug. On the other hand, in the study of Nasr et al. [93] that aimed to enhance colchicine bioavailability and to minimize associated side effects after oral or parenteral administration, cubosomes were proposed for a transdermal delivery of such drug. Following in vivo studies, it was found that transdermal application of colchicine cubosomal gel significantly improves the drug absorption compared with oral solution, with a 4.6-fold greater bioavailability. In addition, Nitha and Siram [94] demonstrated that cubosomes are able to enhance the permeation of dapsone through the skin as compared to marketed formulation and the corresponding free drug solution. Nonetheless, Boge and colleagues [95] evidenced that the antimicrobial peptide LL-37 could be proposed for topical delivery by mean of cubosomes. Particularly, high loadings of peptide within cubosomes induced formation of lamellar phase and a significant bactericidal effect, therefore allowing the possibility to propose cubosomes to increase the local concentration of peptide.

### 2.6. Solid Lipid Nanoparticles (SLNs) and Nanostructured Lipid Carriers (NLCs)

Lipid nanoparticles are carrier systems able to merge the advantages expressed by colloidal lipid emulsions and those of solid matrix particles [22,96,97,98]. Among lipid nanoparticles, solid lipid nanoparticles (SLNs) and nanostructured lipid carriers (NLCs) are colloidal carriers exploiting many effects on skin, and therefore are suitable for skin applications. Figure 5 shows exemplary cryo-TEM images of tristearin SLNs (c) and NLCs (d) [99,100,101]. It has been demonstrated that the solid core of these nanoparticles is able to better sustain and control the release of drug as compared to emulsions or other vesicles, such as liposomes [96].

In particular, SLNs can be achieved at room temperature using solid lipids dispersed in an aqueous surfactant solution able to stabilize the system. On the other hand, NLCs can be obtained from a lipid mixture of solid and liquid phases. Notably, the oily phase is embedded within the solid matrix or placed between the solid particle surface and the surfactant. The inclusion of oily lipids within the solid matrix resulted in the formation of flaws in the nanoparticles crystal lattice that are able to improve the drug loading capacity; however, they also reduce the expulsion of the drug during storage [102,103,104,105].

It has been demonstrated that SLNs display many advantages as delivery systems [103,105]. For instance, SLNs are able to ameliorate the chemical stability of the encapsulated drug due to the protection given by the solid matrix towards hydrolysis and oxidation. Moreover, due to the presence of physiological excipients generally recognized as safe (GRAS), such as glyceryl behenate, glyceryl monostearate, glyceryl palmitostearate, trimyristin, tripalmitin, tristearin, or wax cetylpalmitate, SLNs are acceptable from a cosmetic point of view and well tolerated for skin topical application [106]. Because of their matrix nature, SLNs can be helpful to administrate lipophilic drugs. In addition, SLNs can protect labile agents from deterioration and also modulate the release of loaded drug. The obtaining of these LBNs is allowed by the physico-chemical properties of lipids. Particularly, an emulsification method of the molten lipids followed by re-crystallization was employed. In this way, the use of potentially toxic solvents usually exploited for the preparation of polymeric nanoparticles is avoided. It has to be underlined that to physically stabilize the dispersion, a surfactant (usually in a concentration range between 0.5% and 5%) must be added taking into consideration the type and the concentration of the lipid used for the preparation of LBNs. Notably, polyglycerol methylglucose distearate, sodium cocoamphoacetate, lecithin, poloxamer 188, polysorbate 80, and sucrose fatty acid ester are used for dermal application.

**Figure 6:**
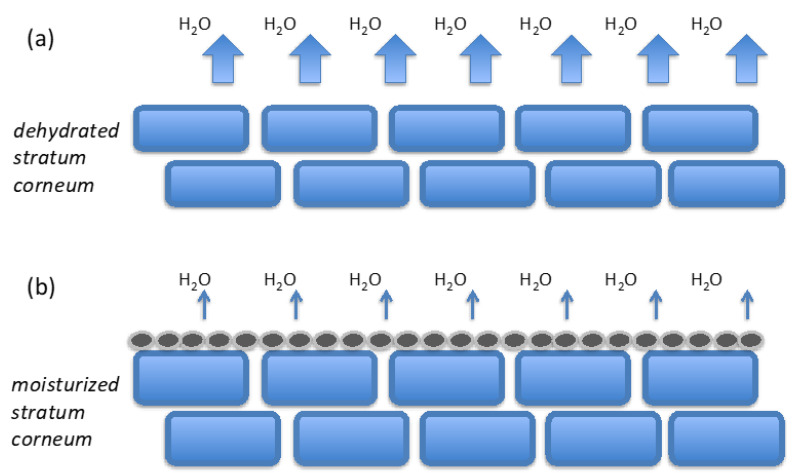
Effect of SLNs or NLCs on TEWL and on skin hydration. (**a**) Water evaporation of untreated skin. (**b**) Occlusion effect of LBNs reducing TEWL.

Although the penetration of these LBNs as whole particles is not reported, SLNs and NLCs show a number of good properties for topical application, such as the high adhesion to the *stratum corneum* that facilitates the penetration of loaded drug within the viable skin [28,30,64]. The enhancement of drug penetration into the different skin layers can be ascribed to an occlusive effect due to the LBN film formation on the skin when the particles size ranges between 200 and 400 nm [22,64,107]. The occlusive properties on the skin displayed by these LBNs ensure the enhancement of the drug penetration thanks to the skin moisture caused by the loss of water after the application of the LBN formulation to the skin. Indeed, this phenomenon induces the lipid particles to form on the skin surface an adhesive layer with consequent occlusion, a reduction of transepidermal water loss (TEWL), and a *stratum corneum* hydration (Figure 6) [11]. The increase of water content within the skin leads to a number of effects such as reduction of eczema symptoms, improvement of healthy human skin, drug permeation increase, and improved uptake of molecules through the skin, as indicated by skin care studies [28,30,108].

SLNs and NLCs appear to be suitable for use on damaged or inflamed skin since they are made of nonirritating and nontoxic GRAS lipids. Usually, these dispersions contain from 5% to 40% of lipids. Taken together, the low lipid content and viscosity of the formulations reduce their suitability for dermal application of drugs. Therefore, the inclusion of these LBNs within a vehicle, such as cream, ointment, or gel, can improve their handling and skin application [91]. However, the possible physical instability, such as dissolution or aggregation of lipid nanoparticles occurring due to the interactions with vehicle constituents, must be considered and evaluated [109]. In this view, the inclusion of SLNs or NLCs dispersion within a hydrogel seems to be a good choice to deliver the drugs as compared to traditional topical and dermatological formulations [110].

The cosmetic use of both loaded and unloaded SLNs has demonstrated that these LBNs are useful to prepare insect repellent formulations and long-lasting perfumes [23], to enhance the uptake of cosmetic agents, the hydration of skin, and to decrease the depth of wrinkles [107], and also to carry UV blockers or UV-absorbing agents enabling the UV protection [3,23]. Moreover, the results obtained in a study concerning the penetration of SLNs into hair follicles indicated a high penetration increase in depth by massaging the scalp after LBNs application [28,111,112,113]. Concerning skin hydration, it was demonstrated that MADs, cubosomes, and SLNs can be used as an alternative to liposomes. In particular, an in vivo corneometer investigation evidenced a more evident skin hydration power of MADs and cubosomes as compared to SLNs and liposomes [113,114].

In literature, many studies investigated the use of SLNs and NLCs as transdermal vehicles for many classes such as antifungals, nonsteroidal anti-inflammatory drugs, glucocorticoids, antihypertensives, or antihistamines. For instance, the anti-inflammatory agent aconitine was studied in order to ameliorate its safety for transdermal administration [115]. It has been found that the thicker the skin region, the faster the drug accumulation, while in perfused skin, the drug content is lower as it passes rapidly to the capillaries. Furthermore, the transdermal permeation of SLNs loaded with aconitine induces an improvement of the anti-inflammatory and analgesic effects on in vivo pain models, together with the efficacy and safety of the drug and a reduction in administration times.

With regard to nonsteroidal anti-inflammatory drugs, a study concerning the treatment of painful and inflamed skin using SLNs, NLCs, and nanoemulsion loaded with lornoxicam is taken as an example [116]. The produced LBNs were physically stable along the 6 months of storage and able to increase the skin penetration rate of lornoxicam with respect to a traditional gel formulation, due to the depot effect providing a sustained release of the drug. The application of hydrogelled SLNs and NLCs containing flurbiprofen or nitrendipine gave rise to similar results [117,118].

The treatment of inflammation via application onto the skin by means of SLNs and NLCs was studied. In one study, the release of three glucocorticoids, namely, prednisolone, diester prednicarbate, and betamethasone 17-valerate, from SLNs and O/W cream was compared [119], indicating that SLNs increase the penetration through the skin of drugs administered topically with respect to the O/W cream, possibly due to the interaction between LBNs and the lipids on the skin surface. Moreover, SLNs and NLCs showed an interesting effect in treatment of allergic skin reactions by prolonging the delivery of administrated antihistaminic drugs via reservoir action, therefore preventing the excess of histamine release [120].

An example of LBNs useful for mucosal administration of clotrimazole is here also reported [100,121]. Particularly, clotrimazole-loaded cubosome or NLC dispersions were compared, either as they are or as included in hydrogels, showing that clotrimazole can be incorporated with high recovery in both LBN types. In addition, NLCs are able to better control drug degradation as compared to cubosomes, probably due to the different nanostructure of LBNs. Indeed, as clearly reported in Figure 4, cubosome dispersion is a miscellany of vesicles and cubic particles, while NLCs are solid particles. However, in vitro experiments on *Candida* cells showed that both types of clotrimazole-loaded LBNs are more active than the free drug. Moreover, both gelled LBN dispersions with poloxamer are able to control the diffusion of the drug.

Lastly, a study concerning the in vivo distribution of the model dye Nile red encapsulated in LBN is reported [122]. Indeed, the mode of drug association with the particle matrix may influence the efficiency of skin targeting. Indeed, it was found that the dye is neither attached to the surface of the particle/surfactant nor located within the micelles nor present in free form but incorporated in the particles or in the shell of the surfactant. This was verified using fluorescence spectroscopy, another physical technique, which provides a better understanding of the type of interaction of the embedded agent with its vector but limited to the fluorescence of the investigated molecule. Therefore, it was possible to discriminate between dye inclusion into the liquid from that inside the solid lipid phase of NLC in which oil droplets are dispersed in a solid lipid matrix or attached to the particle surface.

Next to these LBNs, it has to be mentioned the latest generation of dermal lipid nanoparticles with solid particle matrix, namely SmartLipid [123]. SmartLipids are a mixture of a number (usually up to ten) of structurally different lipids (i.e., mono-, di- and triglycerides, or waxes) with different fatty acid chain lengths that allow the formation of a peculiar “chaotic” and disordered lipid particle matrix able to increase the loading capacity and to firmly include the active agents [123]. It was demonstrated that SmartLipids are able to provide efficient skin penetration of encapsulated drug.

## 3. Conclusions

In these last decades the advancement of nanotechnology and mate-rials science has allowed the development of specialized LBNs suitable for a number of applications. Some LBNs for cutaneous administration, i.e., liposomes, ethosomes, transferosomes, SLNs, NLCs, cubosomes and MADs, have been largely investigated to better understand the interactions of these biocompatible nanosystems with the skin and consequently how drug delivery through the skin occurs. Notwithstanding more research is required to better understand the mechanism of interaction between LBNs and skin, the use biocompatible and biodegradable formulations based on natural lipids able to reduce adverse effects after skin application, can be included among the “green economy” representing sustainable approaches to both producers and consumers. In this overview many studies indicated that LBNs can deeply interact with skin layers, therefore promoting prolonged and effective release for a great deal of drugs.

In addition, this review attempts to summarize and to highlight the possibilities offered by LBN in helping the scientific community to exploit the benefits derived from this kind of nanosystems by looking carefully to the future while also considering scalable manufacturing process, regulatory challenges and costs.

## References

[1] Richardson M. (2003). Understanding the structure and function of the skin. Nurs. Times.

[2] Bouwstra J.A., Ponec M. (2006). The skin barrier in healthy and diseased state. Biochim. Biophys. Acta.

[3] de Souza Guedes L., Martinez R.M., Bou-Chacra N.A., Velasco M.V.R., Rosado C., Baby A.R. (2021). An Overview on Topical Administration of Carotenoids and Coenzyme Q10 Loaded in Lipid Nanoparticles. Antioxidants.

[4] Fore J. (2006). A review of skin and the effects of aging on skin structure and function. Ostomy Wound Manag..

[5] Bouwstra J. (2003). Structure of the skin barrier and its modulation by vesicular formulations. Prog. Lipid Res..

[6] Bukhari S.I., Imam S.S., Ahmad M.Z., Vuddanda P.R., Alshehri S., Mahdi W.A., Ahmad J. (2021). Recent Progress in Lipid Nanoparticles for Cancer Theranostics: Opportunity and Challenges. Pharmaceutics.

[7] Garg A., Sharma G.S., Goyal A.K., Ghosh G., Si S.C., Rath G. (2020). Recent advances in topical carriers of anti-fungal agents. Heliyon.

[8] Korting H.C., Schäfer-Korting M. (2010). Carriers in the topical treatment of skin disease. Handb. Exp. Pharmacol..

[9] Nguyen T., Zuniga R. (2013). Skin conditions: New drugs for managing skin disorders. FP Essent..

[10] Kulkarni V.S., Shaw C. (2016). Formulating Creams, Gels, Lotions, and Suspensions. Essential Chemistry for Formulators of Semisolid and Liquid Dosages.

[11] Araujo V.H.S., Delello Di Filippo L., Duarte J.L., Spósito L., Camargo B.A.F.D., da Silva P.B., Chorilli M. (2021). Exploiting solid lipid nanoparticles and nanostructured lipid carriers for drug delivery against cutaneous fungal infections. Crit. Rev. Microbiol..

[12] Saraceno R., Chiricozzi A., Gabellini M., Chimenti S. (2013). Emerging applications of nanomedicine in dermatology. Skin Res. Technol..

[13] Friedman A., Nasir A. (2011). Nanotechnology and dermatology education in the United States: Data from a pilot survey. J. Drugs Dermatol. JDD.

[14] Papakostas D., Rancan F., Sterry W., Blume-Peytavi U., Vogt A. (2011). Nanoparticles in dermatology. Arch. Dermatol. Res..

[15] DeLouise L.A. (2012). Applications of nanotechnology in dermatology. J. Investig. Dermatol..

[16] Lasoń E. (2020). Topical Administration of Terpenes Encapsulated in Nanostructured Lipid-Based Systems. Molecules.

[17] Kirjavainen M., Urtti A., Valjakka-Koskela R., Kiesvaara J., Mönkkönen J. (1999). Liposome-skin interactions and their effects on the skin permeation of drugs. Eur. J. Pharm. Sci..

[18] Immordino M.L., Dosio F., Cattel L. (2006). Stealth liposomes: Review of the basic science, rationale, and clinical applications, existing and potential. Int. J. Nanomed..

[19] Zhang Z., Wo Y., Zhang Y., Wang D., He R., Chen H., Cui D. (2012). In vitro study of ethosome penetration in human skin and hypertrophic scar tissue. Nanomed. Nanotechnol. Biol. Med..

[20] Touitou E., Alkabes M., Eliaz M. (1997). Ethosomes: Novel vesicular carriers for enhanced skin delivery. Pharm. Res..

[21] Schaferkorting M., Mehnert W., Korting H. (2007). Lipid nanoparticles for improved topical application of drugs for skin diseases. Adv. Drug Deliv. Rev..

[22] Müller R.H., Mäder K., Gohla S. (2000). Solid lipid nanoparticles (SLN) for controlled drug delivery—A review of the state of the art. Eur. J. Pharm. Biopharm..

[23] Jores K., Mehnert W., Drechsler M., Bunjes H., Johann C., Mäder K. (2004). Investigations on the structure of solid lipid nanoparticles (SLN) and oil-loaded solid lipid nanoparticles by photon correlation spectroscopy, field-flow fractionation and transmission electron microscopy. J. Control Release.

[24] Siekmann B., Bunjes H., Koch M.H.J., Westesen K. (2002). Preparation and structural investigations of colloidal dispersions prepared from cubic monoglyceride–water phases. Int. J. Pharm..

[25] Battaglia L., Gallarate M. (2012). Lipid nanoparticles: State of the art, new preparation methods and challenges in drug delivery. Expert Opin. Drug Deliv..

[26] Nyström A.M., Fadeel B. (2012). Safety assessment of nanomaterials: Implications for nanomedicine. J. Control. Release.

[27] Schneider M., Stracke F., Hansen S., Schaefer U.F. (2009). Nanoparticles and their interactions with the dermal barrier. Dermatoendocrinology.

[28] Neubert R.H.H. (2011). Potentials of new nanocarriers for dermal and transdermal drug delivery. Eur. J. Pharm. Biopharm..

[29] Lademann J., Richter H., Schanzer S., Knorr F., Meinke M., Sterry W., Patzelt A. (2011). Penetration and storage of particles in human skin: Perspectives and safety aspects. Eur. J. Pharm. Biopharm..

[30] Prow T.W., Grice J.E., Lin L.L., Faye R., Butler M., Becker W., Wurm E.M.T., Yoong C., Robertson T.A., Soyer H.P. (2011). Nanoparticles and microparticles for skin drug delivery. Adv. Drug Deliv. Rev..

[31] Bolzinger M.-A., Briançon S., Chevalier Y. (2011). Nanoparticles through the skin: Managing conflicting results of inorganic and organic particles in cosmetics and pharmaceutics. Wiley Interdiscip. Rev. Nanomed. Nanobiotechnol..

[32] Bangham A.D., Standish M.M., Watkins J.C. (1965). Diffusion of univalent ions across the lamellae of swollen phospholipids. J. Mol. Biol..

[33] Kapoor B., Gupta R., Gulati M., Singh S.K., Khursheed R., Gupta M. (2019). The Why, Where, Who, How, and What of the vesicular delivery systems. Adv. Colloid Interface Sci..

[34] Zhou X., Hao Y., Yuan L., Pradhan S., Shrestha K., Pradhan O., Liu H., Li W. (2018). Nano-formulations for transdermal drug delivery: A review. Chin. Chem. Lett..

[35] Sala M., Diab R., Elaissari A., Fessi H. (2018). Lipid nanocarriers as skin drug delivery systems: Properties, mechanisms of skin interactions and medical applications. Int. J. Pharm..

[36] Daraee H., Etemadi A., Kouhi M., Alimirzalu S., Akbarzadeh A. (2016). Application of liposomes in medicine and drug delivery. Artif. Cells Nanomed. Biotechnol..

[37] Carita A.C., Eloy J.O., Chorilli M., Lee R.J., Leonardi G.R. (2018). Recent Advances and Perspectives in Liposomes for Cutaneous Drug Delivery. Curr. Med. Chem..

[38] Gupta M., Agrawal U., Vyas S.P. (2012). Nanocarrier-based topical drug delivery for the treatment of skin diseases. Expert Opin. Drug Deliv..

[39] Abu Lila A.S., Ishida T. (2017). Liposomal Delivery Systems: Design Optimization and Current Applications. Biol. Pharm. Bull..

[40] Esposito E., Nastruzzi C., Sguizzato M., Cortesi R. (2019). Nanomedicines to Treat Skin Pathologies with Natural Molecules. Curr. Pharm. Des..

[41] Cevc G., Blume G. (1992). Lipid vesicles penetrate into intact skin owing to the transdermal osmotic gradients and hydration force. Biochim. Biophys. Acta BBA Biomembr..

[42] Cevc G. (1996). Transfersomes, liposomes and other lipid suspensions on the skin: Permeation enhancement, vesicle penetration, and transdermal drug delivery. Crit. Rev. Ther. Drug Carrier Syst..

[43] Hussain A., Singh S., Sharma D., Webster T., Shafaat K., Faruk A. (2017). Elastic liposomes as novel carriers: Recent advances in drug delivery. Int. J. Nanomed..

[44] Carter P., Narasimhan B., Wang Q. (2019). Biocompatible nanoparticles and vesicular systems in transdermal drug delivery for various skin diseases. Int. J. Pharm..

[45] Kumar A., Pathak K., Bali V. (2012). Ultra-adaptable nanovesicular systems: A carrier for systemic delivery of therapeutic agents. Drug Discov. Today.

[46] Elsayed M.M.A., Abdallah O.Y., Naggar V.F., Khalafallah N.M. (2007). Lipid vesicles for skin delivery of drugs: Reviewing three decades of research. Int. J. Pharm..

[47] Lee E.H., Kim A., Oh Y.-K., Kim C.-K. (2005). Effect of edge activators on the formation and transfection efficiency of ultradeformable liposomes. Biomaterials.

[48] Ravikumar P., Tatke P. (2019). Advances in encapsulated dermal formulations in chemoprevention of melanoma: An overview. J. Cosmet. Dermatol..

[49] Abd El-Alim S.H., Kassem A.A., Basha M., Salama A. (2019). Comparative study of liposomes, ethosomes and transfersomes as carriers for enhancing the transdermal delivery of diflunisal: In vitro and in vivo evaluation. Int. J. Pharm..

[50] Cevc G. (2003). Transdermal Drug Delivery of Insulin with Ultradeformable Carriers. Clin. Pharmacokinet..

[51] Schneider-Rauber G., Argenta D.F., Caon T. (2020). Emerging Technologies to Target Drug Delivery to the Skin—The Role of Crystals and Carrier-Based Systems in the Case Study of Dapsone. Pharm. Res..

[52] Ramkar S., Sah A.K., Bhuwane N., Choudhary I., Hemnani N., Suresh P.K. (2020). Nano-Lipidic Carriers as a Tool for Drug Targeting to the Pilosebaceous Units. Curr. Pharm. Des..

[53] Cevc G., Blume G., Schätzlein A., Gebauer D., Paul A. (1996). The skin: A pathway for systemic treatment with patches and lipid-based agent carriers. Adv. Drug Deliv. Rev..

[54] Jadupati M., Amites G., Kumar N.A. (2012). Transferosomes: An opportunistic carrier for Transdermal drug delivery system. Int. J. Pharm. IRJP.

[55] Choi M.J., Maibach H.I. (2005). Elastic vesicles as topical/transdermal drug delivery systems. Int. J. Cosmet. Sci..

[56] Jain S., Patel N., Shah M.K., Khatri P., Vora N. (2017). Recent Advances in Lipid-Based Vesicles and Particulate Carriers for Topical and Transdermal Application. J. Pharm. Sci..

[57] Ijaz H., Qureshi J., Tulain U.R., Iqbal F., Danish Z., Fayyaz A., Sethi A. (2018). Lipid particulate drug delivery systems: A review. Bioinspired Biomim. Nanobiomater..

[58] Rai S., Pandey V., Rai G. (2017). Transfersomes as versatile and flexible nano-vesicular carriers in skin cancer therapy: The state of the art. Nano Rev. Exp..

[59] Ge X., Wei M., He S., Yuan W.-E. (2019). Advances of Non-Ionic Surfactant Vesicles (Niosomes) and Their Application in Drug Delivery. Pharmaceutics.

[60] Muzzalupo R., Tavano L. (2015). Niosomal drug delivery for transdermal targeting: Recent advances. Res. Rep. Transdermal Drug Deliv..

[61] Moghassemi S., Hadjizadeh A. (2014). Nano-niosomes as nanoscale drug delivery systems: An illustrated review. J. Control. Release.

[62] Som I., Bhatia K., Yasir M. (2012). Status of surfactants as penetration enhancers in transdermal drug delivery. J. Pharm. Bioallied Sci..

[63] Paape M.J., Wergin W.P. (1977). The leukocyte as a defense mechanism. J. Am. Vet. Med. Assoc..

[64] Liu M., Wen J., Sharma M. (2020). Solid Lipid Nanoparticles for Topical Drug Delivery: Mechanisms, Dosage Form Perspectives, and Translational Status. Curr. Pharm. Des..

[65] Mohamad E.A., Fahmy H.M. (2020). Niosomes and liposomes as promising carriers for dermal delivery of Annona squamosa extract. Braz. J. Pharm. Sci..

[66] Fang J.-Y., Hong C.-T., Chiu W.-T., Wang Y.-Y. (2001). Effect of liposomes and niosomes on skin permeation of enoxacin. Int. J. Pharm..

[67] Karami M.A., Jalili Rad M., Zadeh B.S.M., Salimi A. (2019). Superoxide dismutase loaded niosomes delivery to hair follicles: Permeation through synthetic membrane and guinea pig skin. Int. J. Appl. Pharm..

[68] Lu B., Huang Y., Chen Z., Ye J., Xu H., Chen W., Long X. (2019). Niosomal Nanocarriers for Enhanced Skin Delivery of Quercetin with Functions of Anti-Tyrosinase and Antioxidant. Molecules.

[69] Touitou E., Dayan N., Bergelson L., Godin B., Eliaz M. (2000). Ethosomes—Novel vesicular carriers for enhanced delivery: Characterization and skin penetration properties. J. Control Release.

[70] Rakesh R., Anoop K.R. (2012). Ethosomes for transdermal and topical drug delivery. Int. J. Pharm. Pharm. Sci..

[71] Godin B., Touitou E. (2003). Ethosomes: New prospects in transdermal delivery. Crit. Rev. Ther. Drug Carrier Syst..

[72] Sguizzato M., Mariani P., Spinozzi F., Benedusi M., Cervellati F., Cortesi R., Drechsler M., Prieux R., Valacchi G., Esposito E. (2020). Ethosomes for Coenzyme Q10 Cutaneous Administration: From Design to 3D Skin Tissue Evaluation. Antioxidants.

[73] Esposito E., Menegatti E., Cortesi R. (2004). Ethosomes and liposomes as topical vehicles for azelaic acid: A preformulation study. Int. J. Cosmet. Sci..

[74] Sguizzato M., Ferrara F., Hallan S.S., Baldisserotto A., Drechsler M., Malatesta M., Costanzo M., Cortesi R., Puglia C., Valacchi G. (2021). Ethosomes and Transethosomes for Mangiferin Transdermal Delivery. Antioxidants.

[75] Zhang Y.-T., Shen L.-N., Wu Z.-H., Zhao J.-H., Feng N.-P. (2014). Evaluation of Skin Viability Effect on Ethosome and Liposome-Mediated Psoralen Delivery via Cell Uptake. J. Pharm. Sci..

[76] Natsheh H., Vettorato E., Touitou E. (2019). Ethosomes for Dermal Administration of Natural Active Molecules. Curr. Pharm. Des..

[77] Touitou E., Godin B., Dayan N., Weiss C., Piliponsky A., Levi-Schaffer F. (2001). Intracellular delivery mediated by an ethosomal carrier. Biomaterials.

[78] Jain S., Tiwary A.K., Sapra B., Jain N.K. (2007). Formulation and evaluation of ethosomes for transdermal delivery of lamivudine. AAPS Pharm. Sci. Tech..

[79] Hallan S.S., Sguizzato M., Drechsler M., Mariani P., Montesi L., Cortesi R., Björklund S., Ruzgas T., Esposito E. (2021). The Potential of Caffeic Acid Lipid Nanoparticulate Systems for Skin Application: In Vitro Assays to Assess Delivery and Antioxidant Effect. Nanomaterials.

[80] Song C.K., Balakrishnan P., Shim C.-K., Chung S.-J., Chong S., Kim D.-D. (2012). A novel vesicular carrier, transethosome, for enhanced skin delivery of voriconazole: Characterization and in vitro/in vivo evaluation. Colloids Surf. B Biointerfaces.

[81] Abdulbaqi I.M., Darwis Y., Khan N.A.K., Assi R.A., Khan A.A. (2016). Ethosomal nanocarriers: The impact of constituents and formulation techniques on ethosomal properties, in vivo studies, and clinical trials. Int. J. Nanomed..

[82] Paiva-Santos A.C., Silva A.L., Guerra C., Peixoto D., Pereira-Silva M., Zeinali M., Mascarenhas-Melo F., Castro R., Veiga F. (2021). Ethosomes as Nanocarriers for the Development of Skin Delivery Formulations. Pharm. Res..

[83] Albash R., Abdelbary A., Refai H., El-Nabarawi M. (2019). Use of transethosomes for enhancing the transdermal delivery of olmesartan medoxomil: In vitro, ex vivo, and in vivo evaluation. Int. J. Nanomed..

[84] Moolakkadath T., Aqil M., Ahad A., Imam S.S., Iqbal B., Sultana Y., Mujeeb M., Iqbal Z. (2018). Development of transethosomes formulation for dermal fisetin delivery: Box–Behnken design, optimization, in vitro skin penetration, vesicles–skin interaction and dermatokinetic studies. Artif. Cells Nanomed. Biotechnol..

[85] Ascenso A., Raposo S., Batista C., Cardoso P., Mendes T., Praça F.G., Bentley M.V.L.B., Simões S. (2015). Development, characterization, and skin delivery studies of related ultradeformable vesicles: Transfersomes, ethosomes, and transethosomes. Int. J. Nanomed..

[86] Gustafsson J., Ljusberg-Wahren H., Almgren M., Larsson K. (1996). Cubic Lipid—Water Phase Dispersed into Submicron Particles. Langmuir.

[87] Esposito E., Ravani L., Mariani P., Contado C., Drechsler M., Puglia C., Cortesi R. (2013). Curcumin containing monoolein aqueous dispersions: A preformulative study. Mater Sci. Eng. C.

[88] Spicer P.T., Hayden K.L., Lynch M.L., Ofori-Boateng A., Burns J.L. (2001). Novel Process for Producing Cubic Liquid Crystalline Nanoparticles (Cubosomes). Langmuir.

[89] Sguizzato M., Mariani P., Ferrara F., Drechsler M., Hallan S.S., Huang N., Simelière F., Khunti N., Cortesi R., Marchetti N. (2020). Nanoparticulate Gels for Cutaneous Administration of Caffeic Acid. Nanomaterials.

[90] Esposito E., Pecorelli A., Sguizzato M., Drechsler M., Mariani P., Carducci F., Cervellati F., Nastruzzi C., Cortesi R., Valacchi G. (2018). Production and Characterization of Nanoparticle Based Hyaluronate Gel Containing Retinyl Palmitate for Wound Healing. Curr. Drug Deliv..

[91] Waghule T., Gorantla S., Rapalli V.K., Shah P., Dubey S.K., Saha R.N., Singhvi G. (2020). Emerging Trends in Topical Delivery of Curcumin Through Lipid Nanocarriers: Effectiveness in Skin Disorders. AAPS Pharm. Sci. Tech..

[92] Peng X., Zhou Y., Han K., Qin L., Dian L., Li G., Pan X., Wu C. (2015). Characterization of cubosomes as a targeted and sustained transdermal delivery system for capsaicin. Drug Des. Devel. Ther..

[93] Nasr M., Younes H., Abdel-Rashid R.S. (2020). Formulation and evaluation of cubosomes containing colchicine for transdermal delivery. Drug Deliv. Transl. Res..

[94] Nithya R., Jerold P., Siram K. (2018). Cubosomes of dapsone enhanced permeation across the skin. J. Drug Deliv. Sci. Technol..

[95] Boge L., Hallstensson K., Ringstad L., Johansson J., Andersson T., Davoudi M., Larsson P.T., Mahlapuu M., Håkansson J., Andersson M. (2019). Cubosomes for topical delivery of the antimicrobial peptide LL-37. Eur. J. Pharm. Biopharm..

[96] Vanic Z., Holaeter A.-M., Skalko-Basnet N. (2015). (Phospho)lipid-based Nanosystems for Skin Administration. Curr. Pharm. Des..

[97] Goyal R., Macri L.K., Kaplan H.M., Kohn J. (2016). Nanoparticles and nanofibers for topical drug delivery. J. Control. Release.

[98] Mehnert W., Mäder K. (2001). Solid lipid nanoparticles: Production, characterization and applications. Adv. Drug Deliv. Rev..

[99] Hallan S.S., Sguizzato M., Esposito E., Cortesi R. (2021). Challenges in the Physical Characterization of Lipid Nanoparticles. Pharmaceutics.

[100] Esposito E., Ravani L., Contado C., Costenaro A., Drechsler M., Rossi D., Menegatti E., Grandini A., Cortesi R. (2013). Clotrimazole nanoparticle gel for mucosal administration. Mater Sci. Eng. C.

[101] Esposito E., Sguizzato M., Drechsler M., Mariani P., Carducci F., Nastruzzi C., Cortesi R. (2017). Progesterone lipid nanoparticles: Scaling up and in vivo human study. Eur. J. Pharm. Biopharm..

[102] Kovacevic A., Savic S., Vuleta G., Müller R.H., Keck C.M. (2011). Polyhydroxy surfactants for the formulation of lipid nanoparticles (SLN and NLC): Effects on size, physical stability and particle matrix structure. Int. J. Pharm..

[103] Uner M. (2006). Preparation, characterization and physico-chemical properties of solid lipid nanoparticles (SLN) and nanostructured lipid carriers (NLC): Their benefits as colloidal drug carrier systems. Die Pharm. Int. J. Pharm. Sci..

[104] Vivek K., Reddy H., Murthy R.S.R. (2007). Investigations of the effect of the lipid matrix on drug entrapment, in vitro release, and physical stability of olanzapine-loaded solid lipid nanoparticles. AAPS Pharm. Sci. Tech..

[105] Souto E.B., Baldim I., Oliveira W.P., Rao R., Yadav N., Gama F.M., Mahant S. (2020). SLN and NLC for topical, dermal, and transdermal drug delivery. Expert Opin. Drug Deliv..

[106] Dobreva M., Stefanov S., Andonova V. (2020). Natural Lipids as Structural Components of Solid Lipid Nanoparticles and Nanostructured Lipid Carriers for Topical Delivery. Curr. Pharm. Des..

[107] Bunjes H., Koch M.H.J., Westesen K. (2000). Effect of Particle Size on Colloidal Solid Triglycerides. Langmuir.

[108] Cheng Y.-C., Li T.S., Su H.L., Lee P.C., Wang H.-M.D. (2020). Transdermal Delivery Systems of Natural Products Applied to Skin Therapy and Care. Molecules.

[109] Westesen K., Bunjes H., Koch M.H.J. (1997). Physicochemical characterization of lipid nanoparticles and evaluation of their drug loading capacity and sustained release potential. J. Control Release.

[110] Liu Y.-C., Lin M.T.-Y., Ng A.H.C., Wong T.T., Mehta J.S. (2020). Nanotechnology for the Treatment of Allergic Conjunctival Diseases. Pharmaceuticals.

[111] Lademann J., Richter H., Teichmann A., Otberg N., Blume-Peytavi U., Luengo J., Weiß B., Schaefer U.F., Lehr C.-M., Wepf R. (2007). Nanoparticles—An efficient carrier for drug delivery into the hair follicles. Eur. J. Pharm. Biopharm..

[112] Knorr F., Lademann J., Patzelt A., Sterry W., Blume-Peytavi U., Vogt A. (2009). Follicular transport route—Research progress and future perspectives. Eur. J. Pharm. Biopharm..

[113] Esposito E., Drechsler M., Mariani P., Sivieri E., Bozzini R., Montesi L., Menegatti E., Cortesi R. (2007). Nanosystems for skin hydration: A comparative study. Int. J. Cosmet. Sci..

[114] Esposito E., Menegatti E., Cortesi R. (2005). Skin care: The innovative nanotechnology to improve the performance of delivery systems. J. Appl. Cosmetol..

[115] Yong-Tai Z., Meng-Qing H., Li-Na S., Ji-Hui Z., Nian-Ping F. (2015). Solid lipid nanoparticles formulated for transdermal aconitine administration and evaluated in vitro and in vivo. J. Biomed. Nanotechnol..

[116] Gönüllü Ü., Üner M., Yener G., Karaman E.F., Aydoğmuş Z. (2015). Formulation and characterization of solid lipid nanoparticles, nanostructured lipid carriers and nanoemulsion of lornoxicam for transdermal delivery. Acta. Pharm..

[117] Bhaskar K., Anbu J., Ravichandiran V., Venkateswarlu V., Rao Y. (2009). Lipid nanoparticles for transdermal delivery of flurbiprofen: Formulation, in vitro, ex vivo and in vivo studies. Lipids Health Dis..

[118] Bhaskar K., Mohan C.K., Lingam M., Mohan S.J., Venkateswarlu V., Rao Y.M., Bhaskar K., Anbu J., Ravichandran V. (2009). Development of SLN and NLC Enriched Hydrogels for Transdermal Delivery of Nitrendipine: In Vitro and In Vivo Characteristics. Drug Dev. Ind. Pharm..

[119] Schlupp P., Blaschke T., Kramer K.D., Höltje H.-D., Mehnert W., Schäfer-Korting M. (2011). Drug Release and Skin Penetration from Solid Lipid Nanoparticles and a Base Cream: A Systematic Approach from a Comparison of Three Glucocorticoids. Skin Pharmacol. Physiol..

[120] Üner M., Karaman E., Aydoğmuş Z. (2014). Solid Lipid Nanoparticles and Nanostructured Lipid Carriers of Loratadine for Topical Application: Physicochemical Stability and Drug Penetration through Rat Skin. Trop. J. Pharm. Res..

[121] Ravani L., Esposito E., Bories C., Moal V.L.-L., Loiseau P.M., Djabourov M., Cortesi R., Bouchemal K. (2013). Clotrimazole-loaded nanostructured lipid carrier hydrogels: Thermal analysis and in vitro studies. Int. J. Pharm..

[122] Lombardi Borgia S., Regehly M., Sivaramakrishnan R., Mehnert W., Korting H.C., Danker K., Röder B., Kramer K.D., Schäfer-Korting M. (2005). Lipid nanoparticles for skin penetration enhancement—Correlation to drug localization within the particle matrix as determined by fluorescence and parelectric spectroscopy. J. Control. Release.

[123] Olechowski F., Müller R.H., Pyo S.M. (2019). BergaCare SmartLipids: Commercial lipophilic active concentrates for improved performance of dermal products. Beilstein J. Nanotechnol..

